# Clinical yield of esophagogastroduodenoscopy and pH-impedance testing in esophageal atresia patients performed according to international guidelines

**DOI:** 10.1093/dote/doaf022

**Published:** 2025-04-03

**Authors:** M van Lennep, C Mussies, M A Benninga, R R Gorter, U Krishnan, M P van Wijk

**Affiliations:** Department of Pediatric Gastroenterology and Nutrition, Emma Children’s Hospital Amsterdam University Medical Center, Amsterdam, The Netherlands; Department of Pediatric Gastroenterology and Nutrition, Emma Children’s Hospital Amsterdam University Medical Center, Amsterdam, The Netherlands; Department of Pediatric Gastroenterology and Nutrition, Emma Children’s Hospital Amsterdam University Medical Center, Amsterdam, The Netherlands; Amsterdam Gastroenterology and Metabolism Research Institute, Amsterdam, The Netherlands; Special Interest Group for Neurogastroenterology, Motility and Gut-Brain Interaction, European Society for Paediatric Gastroenterology, Hepatology and Nutrition, Geneva, Switzerland; Department of Pediatric Surgery, Emma Children’s Hospital Amsterdam University Medical Center, Amsterdam, The Netherlands; Amsterdam Gastroenterology and Metabolism Research Institute, Amsterdam, The Netherlands; Amsterdam Reproduction and Development Research Institute, Amsterdam, The Netherlands; Esophageal Atresia Follow Me Multidisciplinary Program, Emma Children’s Hospital, Amsterdam University Medical Center, location University of Amsterdam, Amsterdam, The Netherlands; Department of Paediatric Gastroenterology, Sydney Children’s Hospital, Sydney, Australia; School of Women’s and Children’s Health, University of New South Wales, Sydney, Australia; Esophageal Atresia Special Interest Group, European Society for Paediatric Gastroenterology, Hepatology and Nutrition, Geneva, Switzerland; Special Interest Group for Neurogastroenterology, Motility and Gut-Brain Interaction, European Society for Paediatric Gastroenterology, Hepatology and Nutrition, Geneva, Switzerland; Department of Pediatric Gastroenterology and Nutrition, Emma Children’s Hospital Amsterdam University Medical Center, Amsterdam, The Netherlands; Amsterdam Gastroenterology and Metabolism Research Institute, Amsterdam, The Netherlands; Esophageal Atresia Follow Me Multidisciplinary Program, Emma Children’s Hospital, Amsterdam University Medical Center, location University of Amsterdam, Amsterdam, The Netherlands; Esophageal Atresia Special Interest Group, European Society for Paediatric Gastroenterology, Hepatology and Nutrition, Geneva, Switzerland; Special Interest Group for Neurogastroenterology, Motility and Gut-Brain Interaction, European Society for Paediatric Gastroenterology, Hepatology and Nutrition, Geneva, Switzerland

**Keywords:** esophageal atresia, esophagogastroduodenoscopy, pH-impedance, surveillance

## Abstract

According to the European Society for Paediatric Gastroenterology Hepatology And Nutrition & North American Society For Pediatric Gastroenterology, Hepatology And Nutrition (ESPGHAN-NASPGHAN) guideline, esophageal atresia (EA) patients should *routinely* undergo esophagogastroduodenoscopy (EGD) with biopsies and/or pH-impedance (pH multichannel intraluminal impedance test; pH-MII) for surveillance purposes. It is additionally recommended to perform these procedures when there is a *clinical indication*: symptoms suggestive of gastroesophageal reflux disease or eosinophilic esophagitis. The aim of this study was to evaluate how often EGD/pH-MII outcomes change management decisions in EA children who come for surveillance and/or for clinical evaluation of their symptoms. A retrospective chart review was conducted of all EA patients who received EGD and/or pH-MII for routine surveillance or because of clinical indication, i.e. symptoms suggestive of gastroesophageal reflux disease or eosinophilic esophagitis. For each procedure, we assessed whether outcomes changed management decisions. Between 2017 and 2020, 41 patients (median age 2.0 [1.0–17.5] years) underwent EGD/pH-MII for surveillance purposes and 64 (3.0 [0.1–15.8] years) for symptom evaluation. Of the 41 patients who underwent surveillance EGD/pH-MII, 18 (43.9%) were *asymptomatic* when interviewed. Eight of these 18 (44.4%) had results that changed management decisions. In total, 23/41 (56.1%) had results that changed management decisions. Sixty-four patients presented clinically with (a combination of) dysphagia (*n* = 50; 78.1%), regurgitation (*n* = 37; 57.8%), heartburn (*n* = 18; 28.1%), and/or respiratory symptoms that were thought to have a gastrointestinal origin (*n* = 24; 37.5%). Results changed management decisions in 34/64 (53.1%) patients who presented with symptoms. There is a high clinical yield of EGD and pH-MII testing in EA patients. More than half of the patients, regardless of indication (routine surveillance or symptom evaluation), had EGD and/or pH-MII results that changed management decisions.

**What is known:**

- Patients with esophageal atresia (EA) often suffer from gastrointestinal symptoms such as dysphagia, regurgitation, and from gastrointestinal comorbidities including gastroesophageal reflux disease (GERD) and/or eosinophilic esophagitis (EoE).
- The ESPGHAN-NASPGHAN consensus-based EA guideline recommends to routinely perform surveillance esophagogastroduodenoscopy (EGD) and pH-impedance (pH-MII) in children with EA to check for these complications and prevent long-term consequences.
- It is further recommended to perform EGD with biopsies and pH-MII procedures when there are symptoms suggestive of GERD or EoE.

**What is new:**

- In EA patients, routine surveillance leads to changes in management decisions in more than half of the patients.
- Even completely *asymptomatic* patients that undergo surveillance procedures have abnormal results that change management decisions in almost half of the cases.
- EGD and pH-MII are also relevant to perform in *symptomatic* patients, as it led to changes in management decisions in more than half of patients.

## INTRODUCTION

Esophageal atresia (EA) is a congenital esophageal anomaly with a worldwide prevalence of ~2.4 per 10,000 births.^1^^,^^2^ After surgical repair, patients commonly suffer from gastrointestinal symptoms and/or comorbidities. These symptoms include dysphagia, regurgitation, feeding difficulties, and/or respiratory symptoms^1^^,^^3–11^ and may be the result of the intrinsically abnormal esophageal motility, esophageal narrowing at the site of the anastomosis, abnormal motility due to gastroesophageal reflux disease (GERD), reflux esophagitis,^8^^,^^12^ or eosinophilic esophagitis (EoE). The latter has been described in up to 17% of EA patients.^13–15^

The international consensus-based ESPGHAN-NASPGHAN guideline on the gastrointestinal and nutritional management of EA patients recommends routine *surveillance* with three esophagogastroduodenoscopies (EGDs) with biopsies during childhood and a pH-impedance (pH multichannel intraluminal impedance test; pH-MII) measurement when acid-suppressive therapy is ceased.^16^ Furthermore, it recommends performing EGD and pH-MII in symptomatic patients to assess the potential underlying gastrointestinal cause of symptoms.^16^

The aim of this study was to evaluate how often EGD and pH-MII outcomes changed management decisions, both in patients who were invited for regular surveillance and in patients who were referred for evaluation of their symptoms.

## METHODS

### Study subjects and study design

All pediatric EA patients (0–18 years) who were invited to undergo EGD and/or pH-MII according to ESPGHAN-NASPGHAN EA guideline recommendations between 2017 and 2020 at the Sydney Children’s Hospital (Sydney, Australia) and Emma Children’s Hospital (Amsterdam, The Netherlands) were eligible.^16^ Patients were prospectively followed according to the guideline recommendations. Clinical data were retrospectively collected.

### Ethical approval

Ethical approval was obtained by the local institutional review board of the Sydney Children’s Hospital. At the Emma Children’s Hospital, the ethical committee exempted this study from formal ethical approval as the Medical Research Involving Human Subjects Act (WMO) did not apply (reference number: W19_336#19.396). According to Dutch legislation, each patient received an opt-out letter. If patients (and/or their caregivers) did not object against the use of their medical data within 6 weeks, patients were included in this study and data collection took place.

### Data collection

Medical charts of included patients were examined for patient characteristics (age, gender, type of EA, associated comorbidities, and clinical symptoms at time of procedure), history of endoscopic and surgical procedures, indication, outcomes of EGD and/or pH-MII, and further management after the procedure.

### Categorization of EGD and pH-MII

EGD and pH-MII procedures were subdivided into two groups:

- Procedures performed for **surveillance** according to guideline recommendations (i.e. routine screening for GERD and EoE).^16^ Patients who were referred for routine surveillance were clinically interviewed. They were classified as being *asymptomatic* when they did not report any symptoms. Patients were scored as *symptomatic* when they reported symptoms during routine surveillance but had not considered visiting a health care professional for it. These symptoms included dysphagia, regurgitation, chest pain, and/or respiratory symptoms with a potential gastrointestinal origin.
- Procedures performed for **evaluation of symptoms** with a (potential) gastrointestinal origin, according to guideline recommendations.^16^ This includes evaluation of dysphagia or feeding difficulties, respiratory symptoms that may have a gastrointestinal cause (e.g. coughing or recurrent chest infections due to suspected laryngeal penetration or micro-aspirations), or symptoms suggestive of GERD or EoE.

If a patient underwent both a surveillance procedure and a procedure for symptom evaluation during the study period, but at different time points, this patient was included twice. However, if symptom evaluation procedures were performed twice, only the first (set of) procedure(s) was included to avoid inclusion bias. Patients who underwent procedures for *follow-up* of previously diagnosed GERD or EoE were also excluded.

### Outcomes of investigations

For each performed EGD and pH-MII, results were collected.^17–22^ See supplemental file 1 for the classification of different procedure results. In short, EGD results included normal results, GERD (microscopic/macroscopic), EoE (>15 eosinophils per high-power field [HPF]), fungal/viral esophageal infections, and/or other macroscopic abnormalities such as hiatus hernia or esophageal narrowing (at discretion of clinician). We categorized pH-MII results into acid exposure time (AET) <3%, 3–6%, 6–9%, or >9%. Additionally, positive symptom index (SI; >50%) and positive symptom association probability (SAP; >95%) were registered in the database.

### Clinical relevance of procedure results

For each performed procedure, we assessed whether outcomes changed management decisions. See Table 1 for an overview of changes in management based on EGD/pH-MII results.

**Table 1:** Overview of changes in management based on EGD/pH-MII outcomes of surveillance tests and tests performed for clinical evaluation of symptoms

**Changes in management decisions**		
	**Surveillance**	**Symptom evaluation**
Normal EGD, normal pH-MII (including negative SAP)	*No change in management*	Reflux symptoms present: – Decision not to start acid-suppressive medication – Decision not to increase acid-suppressive medication – Decision to cease acid-suppressive medicine – Decision to start SSRI for functional symptoms – Referral for additional testing
Normal AET on pH-MII, positive SAP	*No change in management*	– Decision not to start acid-suppressive medication – Decision not to increase acid-suppressive medication – Decision to cease acid-suppressive medicine – Decision to start prokinetic agents – Referral for additional testing
Abnormal AET on pH-MII	– Decision not to cease PPI at age of 12 months – Start PPI – Referral for additional testing	– Referral for additional testing *(note that increasing/adjusting acid-suppressive medication would likely also have been done based on persisting symptoms alone in absence of testing. This was not considered a change in management)*
Abnormal EGD: EoE	– Decision not to cease PPI – Decision to start budesonide	– Decision to start budesonide instead of PPI – Decision to add budesonide to PPI
Abnormal EGD: fungal/viral infection	– Start fungal therapy – Start valaciclovir	– Start other medical therapy instead of PPI
Abnormal EGD: anastomotic narrowing/congenital stenosis	– Dilation (if a surveillance EGD would not have been performed, presence of significant esophageal narrowing would likely not have been detected until deterioration of symptoms would occur in a later stage)	*No change in management*
Abnormal EGD: recurrent TEF	– Referral for surgical procedure	– Referral for surgical procedure

EGD, esophagogastroduodenoscopy; EoE, eosinophilic esophagitis; pH-MII, pH multichannel intraluminal impedance test; PPI, proton pump inhibitor; SAP, symptom association probability; SI, symptom index; TEF, tracheo-esophageal fistula

### Statistical analysis

Statistical analysis was performed using IBM Statistical Package for the Social Sciences (SPSS) for Windows, v 26.0 (Armonk, NY: IBM Corp.). Descriptive data were presented as median [range]. Categorical data were presented as frequencies (%).

Surveillance results were analyzed per procedure and per evaluation timeframes as detailed in the guidelines: EGD: at time of proton pump inhibitor (PPI) cessation, before the age of 10 years and before transition to adulthood, and pH-MII at time of PPI cessation.

Correlations between symptoms and the presence of GERD, EoE, or anastomotic narrowing/stenoses were assessed with Spearman’s rho (*rs*)*.* Strength of the correlation was classified as *rs* = 0.00–0.19, ‘very weak’; *rs* = 0.20–0.39, ‘weak’; *rs* = 0.40–0.59, ‘moderate’; *rs* = 0.60–0.79, ‘strong’; and *rs* = 0.80–1.0, ‘very strong’. A *P*-value of <0.05 was considered statistically significant.

## RESULTS

Between 2017 and 2020, 101 children underwent EGD and/or pH-MII according to guideline recommendations. Two of them objected against the use of their medical data. Eleven patients did not undergo testing even though the guideline would recommend this. In most cases**,** parents chose to opt-out on routine testing as they were not comfortable with anesthesia when their child did not have any symptoms.

In total, 99 children (median 2.5 [0.1–17.5] years; 13% type A, 85% type C, and 2% type E EA) were thus included in this study. Six of them were tested for both surveillance and for symptom evaluation at different time points and were therefore included twice. In total, 41 patients were invited for routine surveillance, and 64 underwent procedures for evaluation of clinical symptoms.

Fifty-eight (58.6%) children were seen at the Sydney Children’s Hospital, Sydney, Australia, and 41 (41.4%) at the Emma Children’s Hospital, Amsterdam UMC, Amsterdam, the Netherlands. Patient characteristics are shown in Table 2.

**Table 2:** Patient characteristics

		**Surveillance, *n* = 41**	**Symptom evaluation, *n* = 64**	**Total, *n* = 105** ^†^
**Male, *n* (%)**		19 (46.3)	41 (64.1)	60 (57.1)
**Age (med, [range])**		2.0 [1.0–17.5]	3.0 [0.1–15.8]	2.5 [0.1–17.5]
**Type of EA, *n* (%)**				
	A	4 (9.8)	9 (14.1)	13 (12.4)
	C	36 (87.8)	54 (84.4)	90 (85.7)
	E	1 (2.4)	1 (1.5)	2 (1.9)
**Associated anomalies, *n* (%)**		20 (48.8)	18 (28.1)	38 (36.2)
	Genetic	0 (0)	4 (6.3)	4 (3.8)
	Vertebral defects	1 (2.4)	4 (6.3)	5 (4.8)
	Anorectal anomalies	6 (14.6)	2 (3.1)	8 (7.6)
	Congenital heart disease	10 (24.4)	6 (9.4)	16 (15.2)
	Renal anomalies	3 (7.3)	3 (46.9)	6 (5.7)
	Limb anomalies	1 (2.4)	3 (46.9)	4 (3.8)
**Clinical symptoms, *n* (%)**		23 (56.1)^‡^	64 (100)	87 (82.8)
	Dysphagia related	21 (51.2)^‡^	50 (78.1)	71 (67.6)
	Regurgitation	12 (29.2)^‡^	37 (57.8)	49 (46.7)
	Heartburn	1 (2.4)^‡^	18 (28.1)	19 (18.1)
	Respiratory symptoms	17 (41.5)^‡^	24 (37.5)	41 (39.0)

EA, esophageal atresia; VACTERL, association of ≥3 of the following congenital abnormalities: vertebral anomalies (V), anal atresia (A), cardiac malformations (C), tracheo-esophageal fistula (TEF), renal dysplasia (R), and limb abnormalities (L).

^†^Five patients were both included in the surveillance as well as symptom evaluation group because they underwent both surveillance and symptom evaluation tests at different time points.

^‡^Children in the surveillance group reported symptoms during their routine follow-up clinical interview. These symptoms were not considered severe enough to visit a health care professional.

### Surveillance procedures

Forty-one patients (median age 2.0 [1.0–17.5] years; 9.8% type A, 87.8% type C, 2.4% type E) came for surveillance (*n* = 13 EGD; *n* = 9 pH-MII; *n* = 19 both procedures). None of the patients was on acid-suppressive therapy during testing (*n* = 20 (48.8%) temporarily ceased acid suppressants for the procedure; *n* = 21 (51.2%) did not take acid-suppressive medication at all).

In 23/41 (56.1%) children, clinically relevant findings on either EGD (*n* = 13/32, 40.6%) and/or pH-MII (*n* = 14/28, 50.0%) resulted in a change in management decisions.

Eighteen of 41 (43.9%) children who came for surveillance did not report any symptoms when clinically interviewed. Of these *asymptomatic* patients, 8/18 (44.4%) had findings that changed management decisions. Table 3a summarizes procedure outcomes for each specifically recommended surveillance moment throughout childhood. Surveillance procedures in children 0–3 years old changed clinical management in 57.7% of cases. Surveillance procedures in children 4–10 years old changed clinical management in 70.0% and in teenagers in 20.0% of cases.

**Table 3a:** Surveillance outcomes per recommended screening moment as recommended by the EA guideline^16^

	**At time of PPI cessation** ^§^ **0–3 years**		**Before adolescence** ^§^ **5–10 years**		**Before transition** ^§^ **15–18 years**		**Overall**	
	***n***	**Changes**	***n***	**Changes**	***n***	**Changes**	***n***	**Changes**
**All tests**	**26**	**15/26 (57.7%)**	**10**	**7/10 (70.0%)**	**5**	**1 (20.0%)**	**41**	**23/41 (56.1%)**
**EGD**	**19**	**6/19 (31.6%)**	**8**	**6/8 (75.0%)**	**5**	**1 (20.0%)**	**32**	**13/32 (40.6%)**
**Normal EGD**	11 (57.9%)	0 (0.0%)	2 (25.0%)	0 (0%)	4 (80.0%)	0 (0.0%)	17 (53.1%)	0 (0.0%)
**Abnormal EGD**	8 (42.1%)	6 (75.0%)	6 (75.0%)	6 (100%)	1 (20.0%)	1 (100%)	15 (46.9%)	13 (87%)
Esophageal narrowing	4 (21.1%)	4 (100%)	2 (25.0%)	2 (100%)	1 (20.0%)	1 (100%)	7 (21.9%)	7 (100%)
GERD	4 (21.1%)	4 (100%)	2 (25.0%)	2 (100%)	0 (0.0%)	0 (0.0%)	6 (18.8%)	6 (100%)
* Macroscopic*	2 (10.5%)^†^	2 (100%)	0 (0.0%)	0 (0.0%)	0 (0.0%)	0 (0.0%)	2 (6.3%)	2 (100%)
*Microscopic*	2 (10.5%)	2 (100%)	2 (25.0%)	2 (100%)	0 (0.0%)	0 (0.0%)	4 (12.5%)	4 (100%)
Erosive lesions at anastomosis	1 (5.3%)	1 (100%)	0 (0.0%)	0 (0%)	0 (0.0%)	0 (0.0%)	1 (3.1%)	1 (100%)
Hiatal hernia	1 (5.3%)	0 (0.0%)	0 (0.0%)	0 (0.0%)	0 (0.0%)	0 (0.0%)	1 (3.1%)	0 (0.0%)
EoE	0 (0.0%)	0 (0.0%)	3 (38%)	3 (100%)	0 (0.0%)	0 (0.0%)	3 (9.4%)	3 (100%)
*Macroscopic*	0 (0.0%)	0 (0.0%)	2 (25.0%)	0 (0.0%)	0 (0.0%)	0 (0.0%)	2 (6.3%)	2 (100%
*Microscopic*	0 (0.0%)	0 (0.0%)	3 (37.5%)	0 (0.0%)	0 (0.0%)	0 (0.0%)	3 (9.4%)	3 (100%)
Fungal hyphae	0 (0.0%)	0 (0.0%)	1 (12.5%)	1 (100%)	0 (0.0%)	0 (0.0%)	1 (3.1%)	1 (100%)
Schatzki ring	1 (5.3%)	0 (0.0%)	0 (0.0%)	0 (0.0%)	0 (0.0%)	0 (0.0%)	1 (3.1%)	0 (0.0%)
Inlet patch	1 (5.3%)	0 (0.0%)	0 (0.0%)	0 (0.0%)	0 (0.0%)	0 (0.0%)	1 (3.1%)	0 (0.0%)
Other	2 (n=1 LA grade A; n=1 LA grade D) (10.5%)^‡^	2 (100%)	0 (0.0%)	0 (0.0%)	0 (0.0%)	0 (0.0%)	2 (6.3%)	2 (100%)
**pH-MII**	**22**	**12/22 (54.5%)**	**4**	**2/4 (50.0%)**	**2**	**0/2 (0.0%)**	**28**	**14/28 (50.0%)**
AET <3%	8 (36.3%)	0 (0.0%)	2 (50.0%)	1 (50.0%)	1 (50.0%)	0 (0.0%)	11 (39%)	1 (9.1%)
AET 3–6%	3 (13.6%)	1 (33.3%)	2 (50.0%)	1 (50%)	1 (50.0%)	0 (0.0%)	6 (21%)	2 (33.3%)
AET 6–9%	3 (13.6%)	3 (100%)	0 (0.0%)	0 (0.0%)	0 (0.0%)	0 (0.0%)	3 (11%)	3 (100%)
AET >9%	8 (36.3%)	8 (100%)	0 (0.0%)	0 (0.0%)	0 (0.0%)	0 (0.0%)	8 (29%)	8 (100%)
SI >50%	6/14 (42.9%)	3 (50.0%)	1/4 (25.0%)	1 (100%)	—	—	7 (25%)	4 (57.1%)
SAP >95%	4/14 (28.6%)	4 (100%)	2/4 (50.0%)	2 (100%)	—	—	6 (21%)	6 (100%)

Surveillance outcomes and changes in management decisions, subdivided into 1.) all test outcomes together; 2. EGD outcomes, either normal or abnormal and 3.) pH-MII outcomes. AET, acid exposure time; EA, esophageal atresia; EGD, esophagogastroduodenoscopy; EoE, eosinophilic esophagitis; GERD, gastroesophageal reflux disease; pH-MII, pH multichannel intraluminal impedance test; PPI, proton pump inhibitor; SAP, symptom association probability; SI, symptom index.

^‡^One patient with inconclusive EoE biopsies and one with Marsh 1 in duodenal biopsies.

^§^Note that age ranges are rather broad due to (1) children who reached the recommended ages for surveillance before the publication of the guideline who were still offered surveillance from 2017 onwards and (2) the COVID-19 pandemic, which resulted in postponement of non-emergent endoscopic procedures at the beginning of the pandemic.

#### Surveillance EGD

Thirty-two patients (median age 2.3 [1.0–17.0] years) underwent EGD for surveillance. Sixteen (50.0%) of them did not report any symptoms when clinically interviewed. The other 16 children did report some symptoms during their routine clinical interview, but they had not considered them severe enough to contact their attending physician. Symptoms included dysphagia (*n* = 15, 46.9%), regurgitation (*n* = 7, 21.9%), and respiratory symptoms (*n* = 11, 34.4%).

Surveillance EGD showed abnormal results in 15/32 (46.9%) which changed management decisions in 13/32 (40.6%) children; see Table 3a for details.

Changed management decisions included (a combination of) the start of medication (*n* = 6), re-start (*n* = 3) or adjustment (*n* = 1) of acid-suppressive therapy while it would normally be ceased, esophageal dilation (*n* = 7), and/or referral for additional testing based on EGD outcome (*n* = 2).

In 6/16 (37.5%) asymptomatic patients, abnormal EGD outcome changed management decisions. This included the start of medication in four children, re-start of acid-suppressive therapy in one child, and referral for additional testing based on EGD results in one child.

##### Correlation of surveillance EGD results and symptoms

Dysphagia and regurgitation symptoms moderately correlated with the presence of esophageal narrowing (*rs* 0.412, *P* = 0.019 and *rs* 0.451, *P* = 0.010, respectively). No correlations were found between presence of symptoms and diagnosis of (both microscopic and macroscopic) esophagitis, elevated AET, or EoE.

#### Surveillance pH-MII

Twenty-eight patients (median age 2.0 [1.0–17.5] years) underwent surveillance pH-MII.

Six of these children (21.4%) did not have any symptoms when clinically interviewed. The other 22 (78.6%) children did report symptoms on clinical interview but had not considered these severe enough to contact a health care professional. Reported symptoms included dysphagia (*n* = 20, 71.4%), regurgitation (*n* = 12, 42.9%), heartburn (*n* = 1, 3.6%), and extra-esophageal symptoms (*n* = 17, 60.7%).

See Table 3a for an overview of procedure results and number of changes in management decisions.

In total, 14/28 (50.0%) patients had surveillance pH-MII outcomes that changed management decisions. This included (a combination of) start medication when it would normally not be started (*n* = 2), continuation (*n* = 7) or adjustment (*n* = 4) of acid-suppressive therapy when it would normally be ceased, and/or referral for additional testing based on pH-MII outcome (*n* = 3).

In 3/6 (50.0%) *asymptomatic* patients, results of pH-MII changed management decisions. This included start with medication (*n* = 2) and adjustment of medication when this would normally be ceased (*n* = 1).

##### Correlation of surveillance pH-MII results and symptoms

Clinical symptoms were not associated with AET, positive SI, or SAP.

### Procedures performed for clinical evaluation of symptoms

Sixty-four children (median age 3.0 [0.1–15.8] years; 14.1% type A, 84.4% type C, 1.5% type E) came for clinical evaluation of symptoms. Thirty-two patients underwent both EGD and pH-MII, 31 underwent EGD testing alone, and one patient underwent pH-MII testing alone.

Symptoms included (a combination of) dysphagia in 50 (78.1%), regurgitation in 37 (57.8%), heartburn in 18 (28.1%), and respiratory symptoms that were thought to have a gastrointestinal origin in 24 (37.5%) children. Thirty-six (56.2%) were on acid-suppressive therapy while they were tested.

**Fig. 1:**
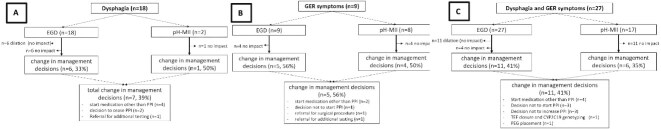
Clinical yield of esophagogastroduodenoscopy (EGD) and 24 h pH-impedance (pH-MII) testing in children that were referred for (A) dysphagia; (B) gastroesophageal reflux (GER) symptoms: regurgitation and/or heartburn; or (C) a combination of GER and dysphagia symptoms. For each performed test, we assessed if test results changed management decisions. Note that esophageal dilations in patients were not considered a change in management, as it is unclear whether these patients would also undergo a dilation based on contrast esophagogram results, if an EGD would not have been performed. EGD, esophagogastroduodenoscopy; PEG, percutaneous endoscopic gastrostomy; pH-MII, pH multichannel intraluminal impedance test; PPI, proton pump inhibitor; TEF, tracheo-esophageal fistula.

In 34/64 (53.1%) children, EGD (*n* = 32/63, 50.8%) and/or pH-MII (*n* = 18/31, 58.1%) outcomes changed management decisions. See Figure 1 for an overview of the clinical yield of EGD and pH-MII in children who were referred for either dysphagia, GER, or a combination of these symptoms.

#### EGD for clinical evaluation of symptoms

Sixty-three children underwent an EGD for evaluation of dysphagia (*n* = 50, 79.3%), regurgitation (*n* = 36, 57.1%), heartburn (*n* = 17, 27.0%), and/or respiratory symptoms with a potential gastrointestinal origin (*n* = 24, 38.1%). Forty-two (66.7%) of 63 patients had abnormal EGD results.

In 32 (50.8%) children, EGD findings, either normal (*n* = 20) or abnormal (*n* = 12), changed management decisions. See Table 3b for an overview of procedure results and number of changes in management decisions.

**Table 3b:** Outcomes of clinical symptom evaluation as recommended by the EA guideline^16^

	**Number of patients (*n*, %)**			**Changes in management decisions**		
	**Overall**	**On PPI**	**Off PPI**	**Overall**	**On PPI**	**Off PPI**
**All tests**	**64**	**36 (56.2)**	**28 (43.8)**	**34/64 (53.1)**	**16/36 (44.4)**	**18/28 (64.3)**
**EGD**	**63**	**35/63 (55.6)**	**28/63 (44.4)**	**32/63 (50.8)**	**15/35 (42.9)**	**17/28 (60.7)**
**Normal EGD**	21 (33.3)	8 (22.9)	13 (46.4)	20/21 (95.2)	7/8 (87.5)	13/13 (100)
**Abnormal EGD**	42 (66.7)	27 (77.1)	15 (53.6)	12/42 (28.6)	8/27 (29.6)	4/15 (26.7)
Stricture/narrowing	26 (41.3)	19 (54.3)	7 (25.0)	0/26 (0)	0/19 (0.0)	0/7 (0.0)
GERD	15 (23.8)	8 (22.9)	7 (25.0)	0/15 (0)	0/8 (0.0)	0/7 (0.0)
*Macroscopic*	1 (1.6)^§^	0 (0)	1 (3.6)	0/1 (0)	0/0 (0.0)	0/1 (0.0)
*Microscopic*	15 (23.8)	8 (22.9)	7 (25.0)	0/15 (0)	0/8 (0.0)	0/7 (0.0)
Erosion at anastomotic site	1 (1.6)	1 (2.9)	0 (0)	0/1 (0)	0/1 (0.0)	0/0 (0.0)
EoE	5 (7.9)	4 (11.4)	1 (3.6)	5/5 (100)	4/4 (100)	1/1 (100)
*Macroscopic*	3 (4.8)	3 (8.6)	0 (0.0)	3/3 (100)	3/3 (100)	0/0 (0.0)
*Microscopic*	5 (7.9)	4 (11.4)	1 (3.6)	5/5 (100)	4/4 (100)	1/1 (100)
TEF	1 (1.6)	1 (2.9)	0 (0)	1/1 (100)	1/1 (100)	0/0 (0.0)
Hiatal hernia	2 (3.2)	0 (0)	2 (7.1)	1/2 (50.0)^‡^	0/0 (0.0)	1/2 (50.0)
Para-esophageal hernia	1 (1.6)	1 (2.9)	0 (0.0)	1/1 (100)	1/1 (100)	0/0 (0.0)
Fungal hyphae	1 (1.6)	0 (0)	1 (3.6)	1/1 (100)	0/0 (0.0)	1/1 (100)
CMV esophagitis	1 (1.6)	1 (2.9)	0 (0.0)	1/1 (100)	1/1 (100)	0/0 (0.0)
Food impaction	2 (3.2)	1 (2.9)	1 (3.6)	0/2 (0.0)	0/1 (0.0)	0/1 (0.0)
Inlet patch	1 (1.6)	0 (0)	1 (3.6)	0/1 (0.0)	0/0 (0.0)	0/1 (0.0)
Diverticulum	2 (3.2)	1 (2.9)	1 (3.6)	2/2 (100)	1/1 (100)	1/1 (100)
**pH-MII**	**31** ^†^	**16/31 (51.6)**	**15/31 (48.4)**	**18/31 (58.1)**	**6/16 (37.5)**	**12/15 (80.0)**
AET <3%	18 (58.1)	8 (50.0)	10 (66.7)	14/18 (77.8)	6/8 (75.0)	8/10 (80.0)
AET 3–6%	8 (25.8)	4 (25.0)	4 (26.7)	3/8 (37.5)	0/4 (0.0)	3/4 (75.0)
AET 6–9%	1 (3.2)	0 (0)	1 (6.7)	0/1 (0.0)	0/0 (0.0)	0/1 (0.0)
AET >9%	4 (12.9)	4 (25.0)	0 (0)	1/4 (25.0)	1/4 (25.0)	0/0 (0.0)
SI >50%	12 (38.7)	10 (62.5)	2 (13.3)	9/12 (75.0)	7/10 (70.0)	2/2 (100)
SAP >95%	15 (48.4)	11 (68.8)	4 (26.7)	10/15 (66.7)	6/11 (54.5)	4/4 (100)

Outcomes of tests performed for clinical symptom evaluation and changes in management decisions, subdivided into 1.) all test outcomes together; 2. EGD outcomes, either normal or abnormal and 3.) pH-MII outcomes. AET, acid exposure time; CMV, cytomegalovirus; EA, esophageal atresia; EGD, esophagogastroduodenoscopy; EoE, eosinophilic esophagitis; GERD, gastroesophageal reflux disease; pH-MII, pH multichannel intraluminal impedance test; PPI, proton pump inhibitor; SAP, symptom association probability; SI, symptom index; TEF, tracheo-esophageal fistula.

^†^In total, 33 patients received pH-MII. Two measurements technically failed, 31 were included in this study.

^‡^In one patient with only a hiatal hernia seen on EGD and two with a diverticulum, management consisted of *not* starting acid-suppressive therapy, which was likely started if management was based on clinical symptoms only.

^§^One patient had both microscopic and macroscopic (grade A/B) esophagitis.

In 12/42 (28.6%) patients with *abnormal* EGD, results changed management decisions. This included (a combination of) start medication when this would normally not be started (*n* = 6), continuation of PPI and referral for additional testing based on EGD results (*n* = 4), referral for surgical procedure (*n* = 3; recurrent tracheo-esophageal fistula (TEF) closure, excision of recurrent stricture, and excision of dilated proximal pouch), and/or dilation of congenital stenosis (*n* = 1).

In the remaining 30 patients with abnormal results, acid-suppressive therapy was continued, adjusted, or increased and/or a dilation was performed. These decisions on management would likely have been similar without testing.

In 21 patients who underwent EGD for symptom evaluation, the results were normal. In 20/21 (95.2%) patients, this outcome changed management decisions. These included the decision *not* to start with acid-suppressive therapy despite symptoms suggestive of GERD (*n* = 14), cease PPI and refer for repeat EGD off-PPI in patients with normal procedure results on medication (*n* = 3), continue same dosage of PPI despite symptoms suggestive of persisting acid-related disease (*n* = 2), and PEG placement for feeding difficulties that could not be attributed to GERD or EoE (*n* = 1).

##### Correlation of EGD results and symptoms

In patients who came for symptom evaluation, dysphagia symptoms weakly correlated with the presence of esophageal narrowing (*rs* 0.348, *P* = 0.005). Additionally, heartburn symptoms negatively correlated with esophageal narrowing (*rs* 0.437, *P* < 0.001). The use of acid-suppressive therapy positively correlated with presence of esophageal narrowing (*rs* 0.324, *P* = 0.010).

No correlations were found between the presence of EoE, microscopic or macroscopic GERD, and symptoms.

#### pH-MII for clinical evaluation of symptoms

Thirty-three patients (median age 9.0 [0.3–15.8] years) underwent a pH-MII for clinical evaluation of symptoms. These included (a combination of) dysphagia (*n* = 23, 69.7%), regurgitation (*n* = 24, 72.7%), heartburn (*n* = 16, 48.5%), and extra-esophageal symptoms (*n* = 16, 48.5%). Sixteen patients (48.5%) were on acid-suppressive medication at time of testing, of which three (9.1%) were also on prokinetics. Two (6.1%) patients temporarily ceased their PPI for diagnostic testing.

Two pH-MII failed (*n* = 1 technical failure, *n* = 1 premature abortion because of intolerance of catheter), and these patients were excluded from further pH-MII analysis. See Table 3 for detailed pH-MII results.

In 18/31 (58.1%) children, pH-MII findings (*n* = 9 normal, *n* = 8 positive symptom association, *n* = 1 elevated AET on PPI) changed management decisions. This included referral for additional testing based on abnormal pH-MII results in two patients (*n* = 1 referral for EGD to rule out EoE and *n* = 1 CYP2C19 genotyping in a child with an AET of 29.0% while on PPI). In seven other patients (*n* = 4 on-PPI), pH-MII showed positive symptom association for nonacid GER episodes, with normal AET. In these patients, management included the start of prokinetic agents or tricyclic antidepressants for hypersensitive esophagus rather than starting acid-suppressive therapy, which would likely have occurred when management was based on symptoms alone. Eight children with normal pH-MII did *not* start with PPI therapy, which would likely have been started based on clinical symptoms alone. Another patient with normal pH-MII was referred for additional testing (laryngobronchoscopy) to assess any potential respiratory cause for its symptoms.

### Eosinophilic esophagitis

In this study, 9/105 (8.6%) children were newly diagnosed with EoE (>15 eosinophils/HPF). Three of them underwent routine surveillance testing (two were asymptomatic and one reported mild dysphagia symptoms at time of EoE diagnosis). Six newly diagnosed patients with EoE came for clinical evaluation of dysphagia (*n* = 5, 83.3%), regurgitation (*n* = 3, 50.0%), chest pain (*n* = 1, 16.7%), and/or respiratory symptoms that were thought to have a gastrointestinal origin (*n* = 4, 66.7%).

Prevalence of EoE in patients who were invited for routine surveillance was 3/41 (7.3%). Prevalence of EoE in patients who underwent procedures for symptom evaluation was 6/64 (9.4%).

In 5/9 (55.6%) children (*n* = 3 symptom evaluation, *n* = 2 surveillance of which one asymptomatic), EGD showed macroscopic signs of EoE according to the EREFS classification.^18^ These included Edema (*n* = 3), Rings (*n* = 1), Exudate (*n* = 4), Furrowing (*n* = 5), and/or Strictures (*n* = 2).

The remaining four (44.4%) children had normal macroscopic EGD results (*n* = 3 symptom evaluation, *n* = 1 surveillance; asymptomatic) and their EoE diagnosis was based on esophageal biopsies with >15 eosinophils per high-power field alone.^23^

## DISCUSSION

This is the largest study to date evaluating the outcome and clinical implications of EGD and pH-MII, performed according to the international EA guideline recommendations.^16^ Our results show that these diagnostic procedures have a high clinical yield both for routine surveillance and for evaluation of symptoms. Based on the results of EGD and/or pH-MII, management decisions changed in more than half of patients overall.

Our results showed that performing EGD and pH-MII is relevant in *symptomatic* patients to assess the etiology of symptoms. Although most common causes for gastrointestinal symptoms in EA patients include the intrinsically abnormal motility and commonly encountered GERD, other gastrointestinal problems, such as EoE, esophageal narrowing, strictures, or esophagitis may cause similar symptoms. Performing EGD and pH-MII according to the guideline recommendation allows for tailored treatment of the underlying problems.

Our results also show that we cannot rely on symptoms alone in this patient group. In over half of *asymptomatic* patients, abnormal test results were found. Although there is debate about the necessity of *surveillance* procedures in EA,^24^ this study clearly shows its benefit, both for endoscopy and pH-MII testing. GERD, EoE, and esophageal narrowing are the most common abnormalities found.

The reason why these children remain asymptomatic may lie in their abnormally developed esophageal innervation which may be further damaged during surgical repair.^12^^,^^25–28^ Abnormal innervation may lead to a decreased esophageal sensitivity. Further studies are needed to test whether this mechanism plays an important role in the absence of symptoms in children with abnormalities found during objective testing.

Furthermore, EA patients may have adjusted their feeding/eating strategies, which could mask their symptoms for parents and caregivers. In fact, if these children habitually use such coping strategies, they may even become less aware or unaware of their symptoms in the long run. If routine surveillance procedures would not be performed, these patients could develop long-term complications of their underlying disease. Early detection of problems in asymptomatic patients allows for timely treatment and may prevent patients from becoming symptomatic.

Our finding of a *negative* correlation between chest pain and esophageal narrowing may be explained by the increased use of PPI in patients with esophageal narrowing which may have reduced chest pain in this patient group. The fact that we found a significantly increased usage of PPI in patients with esophageal narrowing supports the hypothesis^29–31^ that PPIs do not prevent the formation of strictures. However, the time of PPI prescription in relation to stricture formation is not clear based on our data. It might also be that regurgitation symptoms that were caused by esophageal narrowing/stricture might have been mistaken for GER symptoms, which resulted in PPI prescription.

Despite the correlation that was found between esophageal narrowing and dysphagia/regurgitation symptoms, there were no correlations between any symptoms and the presence of EoE, erosive esophagitis, microscopic esophagitis, or pH-MII findings. This is in line with results from previous studies in children with EA.^31–34^ If EGD and/or pH-MII would not be considered a standard procedure when EA patients present with esophageal symptoms, this lack of correlation could result in undertreatment of esophageal pathology. On the other hand, we also show that a significant proportion of children with GER-like symptoms do not have underlying pathology or have abnormalities other than GERD. This finding suggests that there is also a risk of overtreatment with high(er) doses of PPI, as was shown in other studies.^35^^,^^36^ This again underlines the importance of comprehensive gastroesophageal assessment using EGD and pH-MII in case of gastrointestinal symptoms before PPI therapy is empirically started or increased.

It is known that EA patients are at higher risk for developing EoE.^13^^,^^16^ In fact, in our surveillance group, almost 10% of patients were diagnosed with EoE. A higher prevalence of 12% was described during surveillance endoscopy in an older group of EA patients.^37^

Additionally, in our patients who were evaluated for symptoms, EoE was also detected in almost 10%. This is in line with two other studies. One study reported EoE prevalence in EA patients in respectively 1 out of 14 (6.7%) asymptomatic patients and 5/48 (10.4%) symptomatic patients.^38^ Another study calculated an EoE prevalence of 12% in EA patients who underwent surveillance EGD.^37^ In several other studies, with varying EA patient populations, EoE prevalence was even higher compared to our results, ranging from 15% to 30%.^14^^,^^15^^,^^39–41^ Regardless of the exact percentage of patients found in the different studies, the prevalence of EoE in EA seems to be strikingly high compared to a population without EA. Furthermore, in more than a third or our newly diagnosed EoE cases, patients did not have any macroscopic signs of EoE on EGD, which underscores the importance of looking for EoE both in symptomatic and asymptomatic EA patients on EGD as well as with biopsies.

The strengths of our study are that we managed to include a large cohort of EA patients that were prospectively followed according to the guideline recommendations. To our knowledge, this is the largest study assessing EGD and pH-MII results and clinical implications in EA patients.

This study does have some limitations, however. Due to the retrospective collection of data, underreporting of symptoms may have occurred. However, given that both centers involved in this study have multidisciplinary EA clinics that collect data prospectively and are especially focused on detecting EA-related symptoms and comorbidities, we feel that most symptoms will have been registered by the attending physicians. Due to the start of a follow-up outpatient clinic in EA patients in the Netherlands since 2017, where initially only newborns with EA were invited, the age of our cohort is skewed. Although the multidisciplinary outpatient clinic in the Sydney Children’s Hospital started already in 2011, and all age groups were invited at this outpatient clinic, adolescents are underrepresented in our data which is another limitation of this study. Furthermore, this study did not evaluate what the consequences would have been when surveillance procedures would not have been performed. One can hypothesize that asymptomatic or mildly symptomatic patients with abnormal results will become symptomatic at some point, and that treatment can be started when the patient becomes symptomatic. However, the aforementioned poor correlation between symptoms and underlying diseases in this patient population seems a fair argument against such a hypothesis.^27^^,^^28^ Additionally, we believe that it is beneficial to treat patients before they start suffering from symptoms, poor growth, feeding problems, food aversion, or complications of GERD/EoE. Finally, we only included patients who underwent testing, which may lead to selection bias. However, only a small percentage of patients opted out on testing, and no differences between them and our included patients in terms of patient demographics were seen.

## CONCLUSION

In conclusion, results of EGD and/or pH-MII change management decisions in >50% of cases both in symptomatic patients and those who are routinely surveyed. Both normal and abnormal procedure results help to guide management in EA patients.

## Supplementary Material

Supplementary_file_doaf022

## Notes

M. van Lennep and C. Mussies contributed equally as co-first authors

U. Krishnan and M. P. van Wijk contributed equally as co-senior authors

Specific author contributions: Marinde Van Lennep (Conceptualization, Data curation, Formal analysis, Investigation, Project administration, Writing—original draft, Writing—review & editing), Carlijn Mussies (Data curation, Formal analysis, Project administration, Writing—original draft), Marc Benninga (Supervision, Validation, Writing—review & editing), Ramon Gorter (Methodology, Supervision, Writing—review & editing), Usha Krishnan (Conceptualization, Data curation, Investigation, Methodology, Supervision, Writing—review & editing), and Michael van Wijk (Conceptualization, Methodology, Supervision, Writing—original draft, Writing—review & editing).
