# Supplementary material for: Clinical yield of esophagogastroduodenoscopy and pH-impedance testing in esophageal atresia patients performed according to international guidelines
Source: Dis Esophagus. 2025 Apr 3;38(2):doaf022. doi: 10.1093/dote/doaf022 (PMC11997431; doi:10.1093/dote/doaf022)
Supplement: Supplementary_file_doaf022 [file supplementary_file_doaf022.docx]

| **Investigation** | **Outcomes** | | |
| --- | --- | --- | --- |
| **EGD** | Normal | |  |
|  | Abnormal | |  |
|  |  | Macroscopic abnormalities: | |
|  |  |  | Esophageal narrowing / strictures |
|  |  |  | Congenital stenosis |
|  |  |  | Erosive esophagitis including LA classification (17) |
|  |  |  | Erosion at site of the anastomosis |
|  |  |  | Signs of fungal or viral esophageal infection |
|  |  |  | Signs of eosinophilic esophagitis according to the endoscopic EREFS* score (18) |
|  |  |  | Hiatal hernia |
|  |  |  | Para-esophageal hernia |
|  |  |  | Recurrent tracheo-esophageal fistula (TE-fistula) |
|  |  |  | Schatzki ring |
|  |  |  | Inlet patch |
|  |  |  | Diverticulum |
|  |  |  | Food impaction |
|  |  | Microscopic abnormalities | |
|  |  |  | Microscopic signs of reflux esophagitis (19): dilated intercellular spaces/spongiosis, basal cell hyperplasia, papillae elongation |
|  |  |  | EoE (>15 eosinophils / high power field) |
|  |  |  | Barrett esophagus |
| **pH-MII** |  | Total acid exposure time (AET) in % | |
|  |  |  | <3.0% |
|  |  |  | 3.0-5.9% |
|  |  |  | 6.0-8.9% |
|  |  |  | >9.0% |
|  |  | Positive symptom association probability score (SAP, >95% (20)) | |
|  |  | Positive symptom index (SI, >50%, (21)) | |

**Supplemental file 1: classification of test results**
EREFS = edema, rings, exudate, furrows, stricture; LA = Los Angeles; EGD = esophagogastroduodenoscopy; pH-MII = pH Multichannel Intraluminal Impedance test; AET = acid exposure time; SAP = symptom association probability; SI = symptom index; TEF = tracheo-esophageal fistula
